# Ginsenosides Regulates Innate Immunity to Affect Immune Microenvironment of AIH Through Hippo-YAP/TAZ Signaling Pathway

**DOI:** 10.3389/fimmu.2022.851560

**Published:** 2022-02-11

**Authors:** Kehui Zhang, Jiacheng Li, Zhe Shi, Yingying Zhu, Jing Yang, Xiaolin Liu, Renye Que, Liubing Lin, Yirong Chen, Yong Li

**Affiliations:** ^1^ Shanghai Municipal Hospital of Traditional Chinese Medicine, Shanghai University of Traditional Chinese Medicine, Shanghai, China; ^2^ Shanghai University of Traditional Chinese Medicine, Shanghai, China; ^3^ Department of Vascular Surgery, The Seventh People’s Hospital of Shanghai, Shanghai, China

**Keywords:** autoimmune hepatitis, ginsenosides, Hippo-yap, innate immune, immune microenvironment

## Abstract

Autoimmune hepatitis (AIH) is characterized by chronic progressive liver inflammatory, but there is still no safe and effective medicine. Therefore, glucocorticoid remains the top choice for AIH treatment. In previous studies, it has been confirmed that ginsenosides (GSS) can produce glucocorticoid-like effects and therapeutic effects on various autoimmune diseases. However, the mechanism of GSS for AIH remains unclear. As an important part of the innate immune system, bone marrow-derived suppressor cells (MDSC) have been identified as an important driver of follow-up acquired immune response in many autoimmune diseases, including AIH. Herein, it was found out that GSS intervention can be effective in regulating the immune microenvironment and liver impairment induced by Con A in AIH mice. *In vitro*, the MDSCs derived from healthy mice and the T cells deried from AIH mice were co-cultured. Then, different drugs were intervened with to explore the therapeutic mechanism. Besides, the proliferation and differentiation of MDSCs and T cells were analyzed by flow cytometry, while GR, Hippo-YAP signal pathway and the expression of MDSC-related genes and proteins were detected through qRT-PCR and Western Blot. The changes in NO and ROS levels were further analyzed. The trend of related cytokines expression (IFN- γ, TGF- β, IL-10, IL-6, IL-17) was detected by ELISA. Furthermore, an analysis was conducted as to the ALT and liver pathology of mice for evaluating the liver function of mice. It was discovered that MDSCs proliferation was inhibited, and that T cells tended to differentiate into Th17 rather than Treg in AIH mice. Moreover, the intervention of GSS activated GR and Yap, in addition to promoting the proliferation of MDSCs, especially M-MDSCs. This further promoted the differentiation of Treg to enable immune tolerance, thus alleviating liver impairment. Therefore, it was proposed that GSS can alleviate AIH by modulating the innate immunity and adaptive T cell immunity, which may be the underlying mechanism for GSS to mitigate the liver impairment induced by AIH.

## Introduction

Autoimmune hepatitis (AIH) refers to the inflammation of liver parenchyma mediated by autoimmune response (1). The histological features of AIH include interface hepatitis and lymphocyte infiltration, while the serum is characterized by high levels of alanine aminotransferase (ALT), aspartate aminotransferase (AST), immunoglobulin G (IgG) and autoantibodies (2, 3). AIH is mainly found in females, and there are two peaks in the age of onset. One is in childhood and adolescence, and the other is in the 40-60 years old, which can be manifested in different ethnic groups (3–5). At the initial stage of AIH, it can lead to acute hepatitis attack, or occult onset, and the slow progression to liver cirrhosis, hepatocellular carcinoma or death. There are about 35% of AIH patients in the stage of liver cirrhosis (3, 6, 7) at the time of diagnosis. Though the pathogenesis of AIH is still not fully understood, it is certain that this is closely related to heredity and the environment. The current treatment strategy is glucocorticoid alone or in combination with azathioprine or immunosuppressant (8, 9). However, the side effects of these drugs are unbearable to some patients, which restricts their clinical application (10). To sum up, there is still no comprehensive treatment strategy for those patients unfit for the use of corticosteroids and azathioprine (11, 12). Liver transplantation is considered suitable for those patients facing the circumstance that conventional treatment is ineffective or the disease progresses to liver cirrhosis, despite a 5-year recurrence rate (13, 14). Therefore, it is vitally important to explore new treatments and drugs that can used to effectively treat AIH with limited side effects.

The pathological mechanism of AIH involves the destruction of hepatocytes mediated by T lymphocytes, the imbalance of immune regulation and the deficiency of immune response to self or foreign antigens as induced by immune tolerance deficiency, including the imbalance of CD4^+^/CD8^+^T cells, regulatory T cells (Treg cells)/Th17 cells. It ends up leading to immune hyperactivity, autospecific antibodies or cytotoxic T cells attacking autologous hepatocytes, which causes the occurrence and development of AIH (15, 16). In the 1980s, research was conducted to find a group of myeloid-derived inhibitory cells (MDSCs) in tumor patients, which enables tumor cells to evade the immune system by suppressing the immune system (17). In further studies, it was confirmed that MDSCs can significantly inhibit the activation and proliferation of CD4^+^ and CD8^+^T cells, thus promoting their apoptosis (18). MDSCs are capable to accelerate L-arginine metabolism and inhibit T cell proliferation by producing inhibitory factors Arg-1 and iNOS. In addition, such cytokines as TGF- β, IL-1, IL-2 and IL-10 in immune microenvironment can induce MDSCs to activate and release ROS and NO, thus inhibiting T cell-mediated immune response (19). Therefore, it was hypothesized that whether the liver impairment in AIH can be reduced by up-regulating the MDSCs in the immune microenvironment, which may provide a novel solution to AIH treatment.

The elements of Hippo-YAP/TAZ signaling pathway play a significant role in immune regulation. So far, it has been shown in some studies that the increased level of YAP produces the immunosuppressive effects to inhibit not only the activation signals of CD4^+^ and CD8^+^T cells but also the differentiation of Th cells (20). YAP is also considered as essential for the normal function of CD4^+^CD25^+^Treg cells (21). In other studies, it has been confirmed that overactivated Hippo-YAP signals can promote MDSCs recruitment (22). Notably, glucocorticoid is known as a potential activator of YAP (23). As a sort of traditional Chinese medicine, Ginseng has been applied for a long time due to the presence of ginsenosides as a main active ingredient (GSS) (24). GSS shows a structure similar to glucocorticoid steroid, which is conducive not only to up-regulating glucocorticoid receptor (GR) both *in vivo* and *in vitro*, but also to producing glucocorticoid-like effect (25, 26). As suggested by some studies, ginsenoside Rg1 is capable to inhibit not only the secretion of inflammatory cytokines but also the recruitment of CD4^+^ and CD8^+^ T cells to the liver (27). Not only can ginsenoside Rh2 reduce the production of thymic stromal lymphopoietin in mice with atopic dermatitis by inhibiting NF- κ B signal pathway, it is also effective in suppressing the differentiation of immature CD4^+^T cells into Th2 cells and its effect function *in vitro (* 28). Ginsenoside active metabolite M1 can be used to restrict the activation of NLRP3 inflammatory bodies associated with autophagy, regulate the activation of helper T cells, and induce Treg cells production (29). Therefore, it remains debatable whether and how GSS can promote MDSCs proliferation and regulate immune microenvironment, thus protecting the liver through regulation of GR-Hippo-YAP/TAZ signal pathway in AIH.

## Materials and Methods

### Animals

Male specific-pathogen-free (SPF) C57BL/6 mice (4-6 weeks, 20 ± 2 g) were purchased from Shanghai Shrek Experimental Animal Co., Ltd. (Shanghai, China). After purchase, the mice were raised in the animal room without specific pathogens (SPF) in Shanghai Municipal Hospital of Traditional Chinese Medicine. With 7 mice in each cage, they were allowed free access to foods and drinks. Throughout one week when the mice were fed adaptively, the temperature was maintained at 20-25 °C and the humidity was restricted to the range of 40-70%.

### Con A-Induced AIH Model Mice and Treatment

The mice were injected with the IV Con A (Sigma Chemical, USA) dissolved in PBS *via* the tail vein (20 mg/kg).

To verify the effect of MDSCs proliferation on AIH liver injury, the spleen MDSCs (about 5*10^6^cells/mouse) isolated using immunomagnetic beads or MDSCs antagonist Gr-1 (Biolegend, USA, 100μg/mouse) were infused into the mice *via* caudal vein for 3 days before Con A injection, respectively, thus resulting in the changes to MDSCs load in AIH mice. Besides, 1640 culture medium was used for the control group. One hour after the last administration, Con A was injected into the tail vein of the mice. With the elapse of another 12 hours, the mice were killed.

In order to investigate the protective effect of Hippo-YAP pathway against Con A-induced liver impairment, both YAP inhibitor Verteporfin (VP, MCE, USA, 100mg/kg) and agonist Doxycycline (Doxy, MCE, USA, 50mg/kg) were injected intraperitoneally every other day at the beginning of the week before Con A injection, respectively, while the control group was injected with the same dose of normal saline.

To verify the regulatory effect of Dexamethasone (DEX, Sigma, USA, 5mg/kg) and GSS (Yuanye, Shanghai, China, 50mg/kg) on Hippo-YAP signaling pathway and immune microenvironment for AIH mice, the mice were treated with DEX, GSS and glucocorticoid inhibitor Mifepristone (RU-486, sigma, USA, 2mg/kg), respectively. Within one week before the injection of Con A *via* tail vein, the mice were subjected to the intraperitoneal injection of DEX or RU or intragastric administration of GSS on a daily basis. Then, Con A was injected into the tail vein on day 7. Finally, the mice were killed 12 hours later.

With retrobulbar blood taken, some of them were centrifuged for 3000g for 10 minutes, while the serum was retained for ALT detection and enzyme linked immunosorbent assay (ELISA). In the meantime, some of them were anticoagulated with heparin for later analysis by flow cytometry. The liver was taken, part of which was soaked in tissue fixation solution for follow-up pathological examination. Meanwhile, part of it was placed in -80°C or liquid nitrogen tank, followed by WB, PCR, etc. Then, the spleen was taken and ground for flow analysis, or magnetic beads were used to separate MDSCs and T cells. The bone of the lower limb was removed and the bone marrow was flushed out for flow identification.

### Isolation and Proliferation of T Cells

First of all, Con A was injected into the tail vein of C57 mice and the mice were killed 12 hours later to remove the spleen. With the MojoSort™ mouse CD3 T Cell Isolation Kit (Biolegend, USA) used to enrich CD3^+^T cells from splenic mononuclear cells through the magnetic bead negative selection method, the purity of the cells exceeded 90%. Then, the sorted T cells were suspended in a 96-well plate coated with anti-mouse CD3 (Biolegend, USA, 3ug/ml) and anti-mouse CD28 (Biolegend, USA, 3ug/ml) cytokines, so as to ensure that the number of cells in each well would fall within the range of 3×10^5^~5×10^5^cells. Besides, IL-2 (Biolegend, USA, 50ng/ml) was added into the well for the proliferation of T cells *in vitro* for 72 hours. Finally, the medium was half changed after 3 days of culture, and the cells were obtained on day 5.

### Isolation and Proliferation of MDSCs

The spleen of healthy mice was isolated and instilled into splenic single cell suspension. With the assistance of Myeloid-Derived Suppressor Cell Isolation Kit (Mouse) (Miltenyi Biote, Germany), MDSCs cells were enriched by means of two positive magnetic beads sorting (enriching Gr-1^high^Ly6G^+^cells and Gr-1^dim^Ly6G^-^cells, respectively). Including GM-CSF (PeproTech, USA, 10ng/mL) and IL-6 (PeproTech, USA, 10ng/mL), cytokines were added after resuspension with complete culture medium, while the cells were cultured in cell incubator for 48 hours.

### Co-Culture of MDSCs and T Cells in Transwell Chamber

To demonstrate the effect of MDSCs on T cells function, MDSCs and T cells were co-cultured in Transwell chamber. Obtained by following the above process, the MDSCs and T cells were co-cultured in the Transwell chamber at a 1:1 ratio for 24 h. According to the exact experimental purpose, there were different drugs added to the culture system: Doxy (MCE, USA, 4µg/mL), VP (MCE, USA, 20nmol/L), DEX (Sigma, USA, 0.1µmol/L), RU (Sigma, USA, 1µmol/L), GSS (Yuanye, Shanghai, China, 1mg/ml) and GSS+RU.

### Flow Cytometry

For the characterization of MDSCs, the freshly-isolated MDSCs and single cell suspension of spleen, born marrow and peripheral blood were surface-stained using the following Abs: CD11b, Gr-1, Ly6C and Ly6G. In addition, isolated T cells were surface-stained with CD4 and CD25, before the treatment with eBioscience™ FOXP3/Transcription Factor Staining Buffer Set (Invitrogen, USA). Furthermore, FOXP3 and IL-17 were applied for intracellular and nuclear staining, respectively. Finally, it was analyzed using flow cytometry.

### ALT Detection

The mice were anesthetized through 100mg/kg ketamine intraperitoneal injection, while blood and liver samples were collected for subsequent biochemical analysis. The pre-treatment of serum ALT detection samples was carried out as follows. The whole blood was collected using 1.5ml tip-bottom EP tube, placed at room temperature for 1 hour, and then centrifuged under the conditions of 3000×g, 10 minutes and 4 °C. The supernatant was collected into a new EP tube for storage at-80 °C. ALT and AST kits (Nanjing Jiancheng Biology Research Institute Co., Ltd.) were employed to determine the level of serum.

### Reactive Oxygen Species (ROS) Quantification

In liver tissue: Briefly, 200 mg of liver tissue was homogenized in 2 mL of ice-cold Tris-HCl buffer (Beyotime, China, 40mM, pH=7.4). The solution of tissue homogenate (100μL) and 1 mL 2’,7’-dichlorofluorescein diacetate (DCFH-DA, Invitgen, USA, 10μM) (diluted with 1:1000 in Tris-HCl buffer) was incubated at 37°C for 40 minutes, and the fluorescence value was determined using a VictorX3 multiplate reader (λex = 485 nm and λem = 525 nm).

ROS expression in MDSCs: The oxidation-sensitive dye (DCFH-DA) was diluted with a serum-free culture medium at a ratio of 1:1000 to the final concentration of 10μmol/L. Then, the cells were collected and suspended in diluted DCFH-DA, incubated in a cell incubator at 37 °C for 20 minutes, and mixed upside down at a 5-minute interval. The cells were washed with serum-free cell culture medium for three times, so as to completely remove the DCFH-DA failing to enter the cells. After the collection of cells, the fluorescence value was determined through flow meter FITC channel.

### NO Level Detection

The content of NO in serum and supernatant was determined in line with the instruction from NO detected Kit. Finally, the absorbance was recorded at 540 nm by a plate reader.

### Cytokine ELISA

Whole blood and supernatant were collected while the serum was isolated through centrifugation. The levels of serum and supernatant of INF-γ, TGF-β, IL-10, IL-6 and IL-17 were determined using ELISA kits (R&D Systems).

### Protein Extraction and Western Blot Analysis

After being extracted from liver tissues and cells, the proteins were electrophoresed on SDS-Page, and then electroblotted onto polyvinylidene difluoride membrane. After being blocked with 5% Bull Serum Albumin (BSA), the membrane was incubated with the primary antibodies, and then with appropriate secondary horseradish-peroxidase-labeled antibody (1:2000; CST signaling, USA). The specific bands were detected by means of enhanced chemiluminescence assay. As for primary antibodies and their working concentrations, they were listed in the supplementary reagent.

### RNA Isolation and qRT-PCR

Trizol (Invitrogen,USA) was applied to carry out total RNA extraction. Then, reverse transcription and quantitative real-time PCR were performed using PrimeScript^®^ RT reagent kit (Takara, Japan) and SYBR Premix Ex Taq (TaKaRa, Japan), respectively. The production of primers was supported by Shanghai Shenggong Biology Co., Ltd. The 2-ΔΔCt method was adopted to calculate the expression levels of genes normalized to GAPDHA levels in each sample. The primer sequences were indicated in supplementary reagent.

### Histopathology

Cut from paraffin-embedded liver tissue, sections (4μm) were fixed in formalin (Beyotime, China), and then stained with haematoxylin and eosin (H&E). Finally, the severity of liver impairment was evaluated.

### Statistical Analysis

The research results were analyzed and plotted using SPSS22.0, ImageJ and GraphPad5.0 software. The data were indicated as mean ± SEM (Standard Error of Mean). Additionally, the one-way analysis of variance (one-way ANOVA) was conducted to draw comparison between groups, with P < 0.05 treated as statistically significant.

## Results

### Injection of MDSCs Into AIH Mice Induced by Con A Can Alleviate Inflammation and Immune Injury

**Figure 1:**
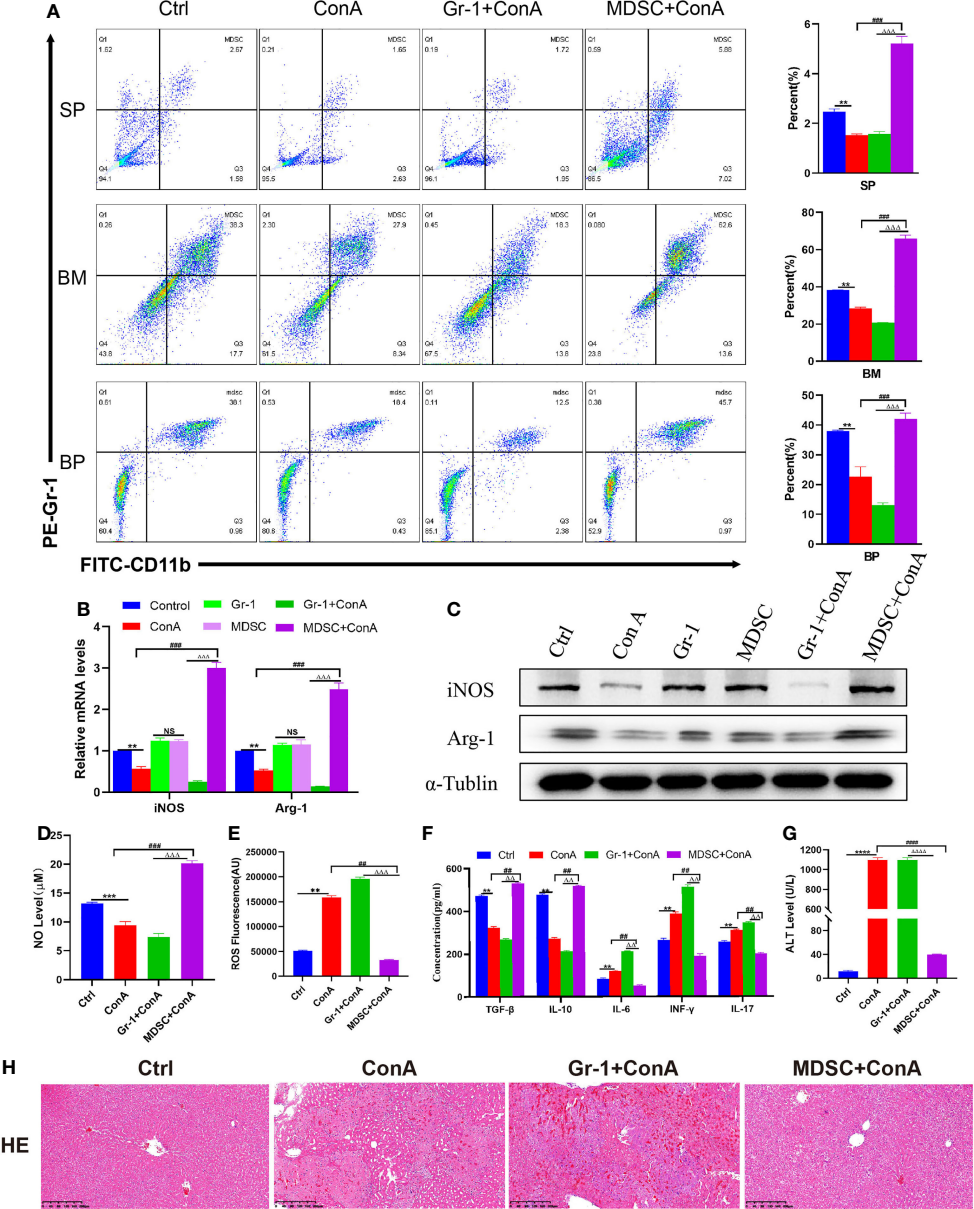
Injection of MDSCs into AIH mice induced by Con A can alleviate inflammation and immune injury. **(A)** The frequencies of MDSCs in spleen, bone marrow (BM) and peripheral blood (BP) (n=3). **(B)** Expression of related genes was evaluated by qRT-PCR in liver (n=3). **(C)** The relative expression levels of iNOS and Arg-1 protein in liver tissue were detected by Western blot (n=3). **(D)** No concentration in liver tissue (n=3). **(E)** Concentration of ROS in liver tissue(n=3). **(F)** The levels of related cytokines in liver tissue were detected by ELISA (n=3). **(G)** Serum ALT level (n=7). **(H)** Representative H&E histopathology staining of liver tissue (×100; scale bar: 200μm) (n=3). Data are expressed in mean ± SEM; **P < 0.01, ***P < 0.001, ****P < 0.0001, control group versus Con A group; ##P < 0.01, ###P < 0.001, ####P < 0.0001, Con A group versus MDSCs +Con A group; △△P < 0.01, △△△P < 0.001, △△△△P < 0.0001, NS= no significant difference. Gr-1 +Con A group versus MDSCs+ Con A group; NS, not significant.

A Con A-induced AIH model was established to evaluate the inflammatory damage and changes to immune damage in experimental AIH mice. In the course of flow cytometry analysis, the frequencies of MDSCs showed a decreasing trend in Con A-treated mice compared to the control mice in the spleen, bone marrow (BM), and peripheral blood (PB) (**Figure 1A**). Inducible nitric oxide synthase (iNOS) and arginase-1 (Arg-1) were found to be involved in the immunosuppressive regulation of MDSCs (30). Through qRT-PCR assays, it was discovered that the transcript levels of iNOS and Arg-1 were reduced in the liver tissues of those mice treated with Con A, when compared to normal controls. Western blot experiments were also conducted to confirm the same trend of expression (**Figure 1C**). As mentioned above, there were two main subsets of MDSCs identified, namely, the granulocyte subset(G-MDSC) and the monocyte subset(M-MDSC). The G-MDSCs show high levels of ROS and low levels of NO, while the monocyte subpopulations display low levels of ROS and high levels of NO, with both subpopulations expressing Arg-1 (31). It can be seen from above that ROS is another significant indicator of tissue oxidative damage (32). In this study, the levels of NO and ROS in liver tissue were determined to find out that the level of NO in the liver tissue of Con A-treated mice was significantly reduced compared to the normal control group while the level of ROS was significantly increased (**Figures 1D, E**). At the same time, the levels of serum IL-6, INF-γ and IL-17 were significantly increased after Con A treatment compared to the control group, while the levels of cytokines IL-10 and TGF-β that could mediate immunosuppressive effects were reduced (**Figure 1F**). In addition, the serum ALT level of mice was significantly increased in the Con A-induced immune-mediated hepatitis model (**Figure 1G**). Focal hepatocyte necrosis was observed by H&E staining of liver tissue, while extensive inflammation infiltration was observed in portal vein area and around central vein (**Figure 1H**). With Gr-1 injected into the tail vein of mice to deplete the MDSCs in the mice, the above-mentioned indicators were detected. According to the results, the immunosuppression and inflammatory damage caused to the mice were more serious. To further verify whether MDSCs are capable to alleviate liver damage and enhance immune tolerance for AIH mice, MDSCs were reinfused from the healthy mouse spleens sorted by magnetic beads into mice. Compared with Con A and Gr-1 treated groups, iNOS and Arg-1 expression levels were found to increase in liver tissue (**Figures 1B, C**). Besides, NO and ROS levels were detected, suggesting the rise of NO levels and the decline of ROS expression (**Figures 1D, E**). Then, the expression of peripheral blood-related cytokines was examined. After the treatment by MDSCs, the level of inflammatory cytokine IL-6 dropped but the expression of immunosuppression-related cytokines IL-10 and INF-r was enhanced, thus promoting the proliferation of Treg cells. In contrast, the pro-inflammatory immune factors IL-17 and TGF-β were reduced, thus inhibiting TH17 cell differentiation. In addition, the mice pretreated with MDSCs showed resistance to Con A-induced liver impairment, as evidenced by the diminishing liver enzyme levels, reduced liver tissue congestion, edema, and vacuolar necrosis, as well as a clear liver structure (**Figures 1G, H**). To sum up, these results demonstrate that MDSCs can produce a protective effect against Con A-induced AIH. The infusion of MDSCs suppressed the inflammatory response in mice and reduced the immune damage caused to the liver.

### The Hippo-YAP Signal Pathway Regulates the Innate Immune, Reshapes the Immune Microenvironment and Inhibits Immune Liver Injury in Mice

**Figure 2:**
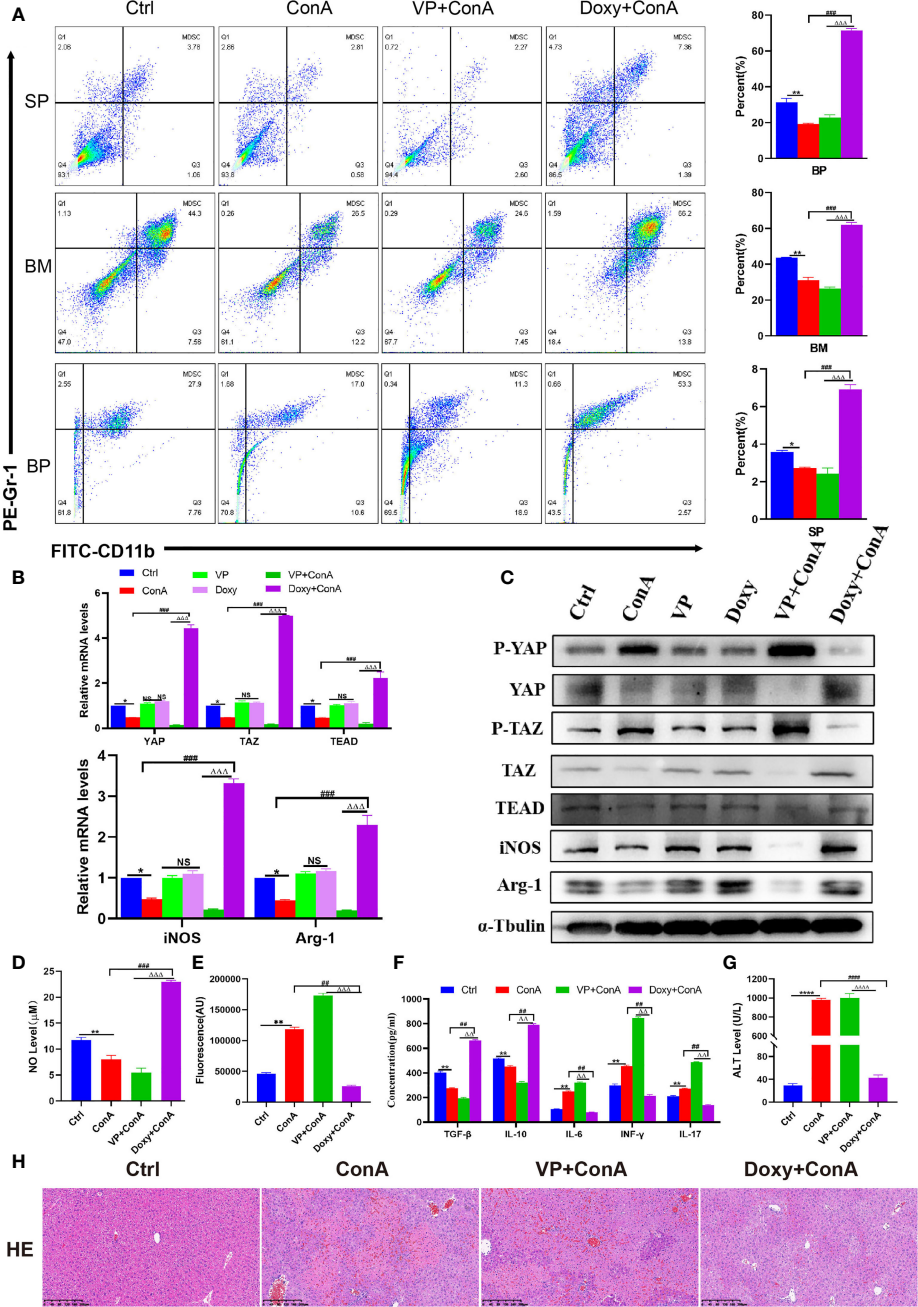
The Hippo-YAP signal pathway regulates the innate immune, reshapes the immune microenvironment and inhibits immune liver injury in mice. **(A)** The frequencies of MDSCs in spleen, bone marrow(BM) and peripheral blood(BP)(n=3). **(B)** Expression of related genes was evaluated by RT-PCR in liver (n=3). **(C)** The relative expression levels of related protein in liver tissue were detected by Western blot (n=3). **(D)** No concentration in liver tissue(n=3). **(E)** Concentration of ROS in liver tissue(n=3). **(F)** The levels of related cytokines in liver tissue were detected by ELISA (n=3). **(G)** Serum ALT level(n=7). **(H)** Representative H&E histopathology staining of liver tissue (×100; scale bar: 200μm) (n=3). Data are expressed in mean ± SEM; *P < 0.05, **P < 0.01, ****P < 0.0001, control group versus Con A group; #P < 0.05, ##P < 0.01, ###P < 0.001, ####P < 0.0001, Con A group versus Doxy +Con A group; △△P < 0.01, △△△P < 0.001, △△△△P < 0.0001, VP +Con A group versus Doxy+ Con A group; NS, no significant difference.

The activation of MDSCs involves a number of different signaling pathways, including STAT6, STAT1, and nuclear factor-κB (NF-κB), etc (33). In some recent studies, it has been suggested that the activation of the Hippo-YAP signaling pathway can promote the expression and secretion of various cytokines/chemokines, thereby enhancing the differentiation and accumulation of MDSCs both *in vivo* and *in vitro (* 22). Since YAP/TAZ is the core effector of the Hippo pathway, the most reasonable strategy for the Hippo pathway is through the transcriptional co-activators YAP/TAZ and TEADs. In the AIH mouse model induced by Con A, it was found out that the expression of YAP, TAZ and TEAD declined, while that of phosphorylated YAP/TAZ was improved (**Figures 2B, C**), indicating the activation of the Hippo pathway. Besides, the phosphorylated YAP will bind to 14-3-3 protein, continue in the cytoplasm, and get degraded by the ubiquitin-dependent proteasome (34). The mice were pretreated with the YAP agonist Doxycycline (Doxy) and the inhibitor Verteporfin (VP) to demonstrate whether activating the Hippo-YAP signaling pathway can produce an immunosuppressive effect by promoting the proliferation and differentiation of MDSCs. Firstly, flow cytometry analysis was conducted to reveal that the proportion of MDSCs in the Doxy group was increased whether in the spleen, bone marrow or peripheral blood, despite a decrease in the VP group (**Figure 2A**). Meanwhile, it was found out from qRT-PCR detection that the expression of YAP, TAZ and TEAD genes was suppressed in the liver tissue of mice from the VP treatment group. Similarly, the expression of downstream genes of MDSCs (iNOS and Arg-1) also show a decreasing trend (**Figure 2B**). In addition, WB detection was performed to show an increase in the expression of phosphorylated YAP and TAZ, which confirms that the inhibition of YAP/TAZ-TEAD complex after VP administration can further inhibit the expression of downstream molecules in MDSCs (**Figure 2C**). In the Doxy group, the expression of p-YAP and p-TAZ protein diminished in liver tissues. Then, unphosphorylated proteins were transferred to the nucleus and combined with TEAD, thus producing target gene effects (**Figure 2C**). In the Doxy group, there was an increase shown in the expression of iNOS and Arg-1 mRNA and protein levels (**Figures 2B, C**). In the meantime, the expression level of NO in liver tissue was detected with a microplate reader, which confirms the increased level of NO expression in the Doxy group. On the contrary, the intervention of VP inhibited the production of NO to a significant extent (**Figure 2D**). Furthermore, the fluorescence quantitative detection of ROS suggested that ROS expression was inhibited in liver tissue after the up-regulation of YAP/TAZ expression (**Figure 2E**). Then, a test was conducted on the expression of related cytokines in peripheral blood (**Figure 2F**). After Doxy treatment, the level of inflammatory factor IL-6 declined, while the expression of immunosuppression-related cytokines IL-10 and INF-r was improved, thus promoting the proliferation of Treg cells. On the contrary, there was a rise in the pro-inflammatory immune factors IL-17 and TGF-β, which promoted the differentiation of Th17 cells. In addition, different from the model group, the mice pretreated with Doxy showed resistance to Con A-induced liver impairment, with a reduction in the level of liver enzymes. Besides, liver pathology suggested a decrease in hepatic hyperemia, edema and vacuolar necrosis, while the liver structures were undamaged (**Figures 2G, H**). On the contrary, the application of YAP inhibitors aggravated liver impairment, as characterized by a significant increase in liver enzymes, an increase in the area of focal hepatocyte necrosis, the extensive infiltration of inflammatory cells, and the destruction of liver structures (**Figures 2G, H**). To sum up, after the activation of YAP/TAZ-TEAD signal pathway, the proportion of MDSCs increased *in vivo*, which promoted the expression of iNOS and NO, inhibited oxidative stress injury, produced an immunosuppressive effect, and prevented hepatocyte necrosis.

### The Activation of YAP Promotes MDSCs Proportion and Immunosuppression *In Vitro*

**Figure 3:**
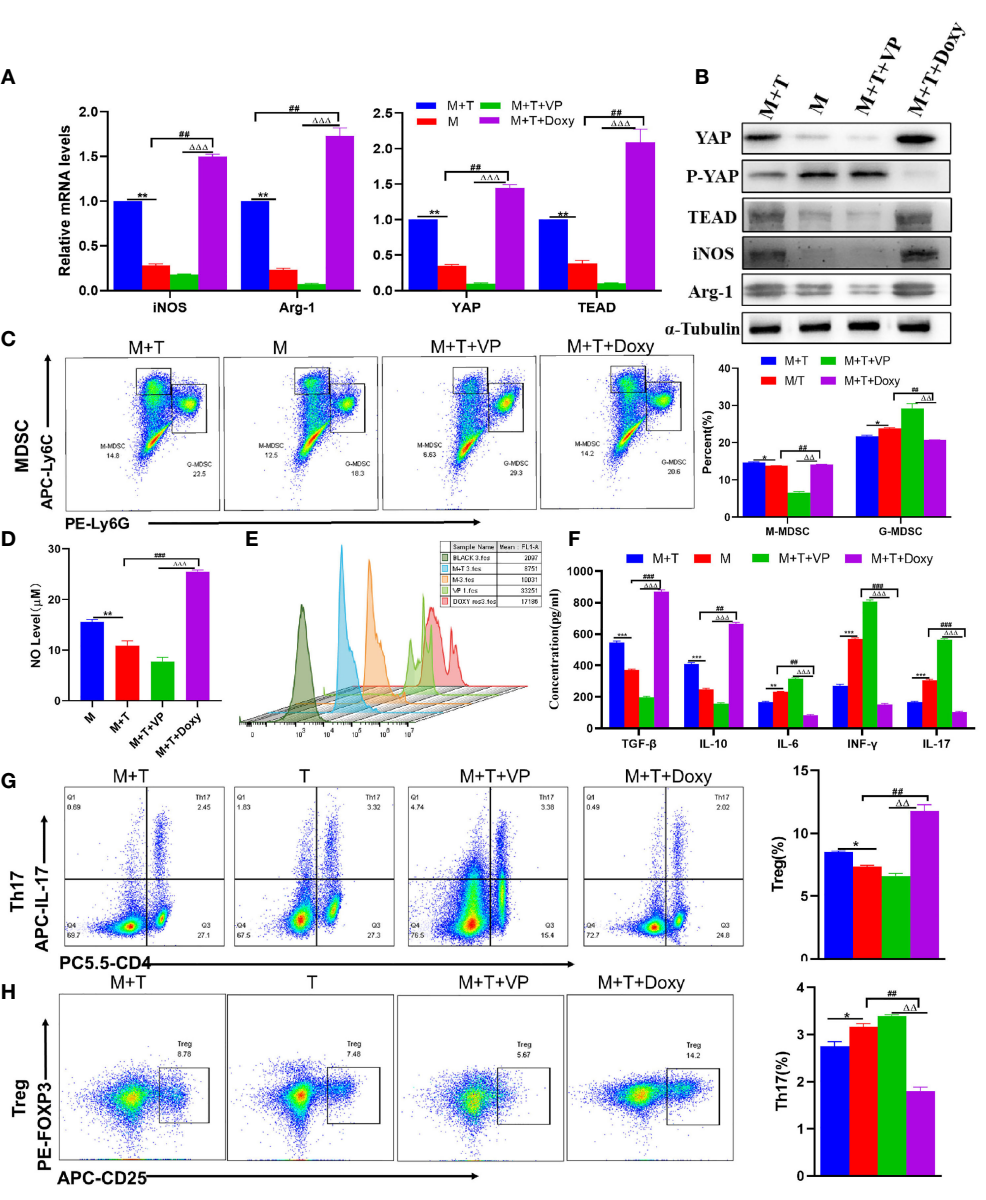
The activation of YAP promotes MDSCs proliferation and immunosuppression *in vitro*. **(A)** Expression of related genes was evaluated using qRT-PCR; **(B)** Levels of related protein expression in MDSCs were analyzed by Western blotting; **(C)** Frequencies of M-MDSC (CD11b^+^Ly6C^high^ Ly6G^-^) and G-MDSC (CD11b^+^Ly6C^low^Ly6G^+^); **(D)** Detection of NO level in supernatant of cells; **(E)** ROS level measured in MDSCs using DCFH-DA staining; **(F)** The production of IFN-γ, TGF-β, IL-10, IL-6, IL-17 in the supernatants was measured by ELISA; **(G)** Frequencies of Tregs (CD4^+^CD25^+^Foxp3^+^); **(H)** Frequencies of Th17 (CD4^+^IL-17^+^); n=3. Data are expressed in mean ± SEM;. *P < 0.05, **P < 0.01, ***P < 0.001, M/T group versus M+T group; ##P < 0.01, ###P < 0.001, M/T group versus M+T+ Doxy; △△P < 0.01, △△△P < 0.001, M+T+ Doxy versus M+T+VP group.

Given the above data, it was speculated that the up-regulation of YAP expression may play a role in regulating the differentiation of Tregs and Th17 cells through the enhanced MDSCs activation. To validate this hypothesis, the MDSCs derived from healthy mice and T cells derived from AIH mice were co-cultured at a ratio of 1:1; with Doxy or VP added to MDSCs and T cells co-culture system respectively. First of all, it was confirmed by qRT-PCR and WB detection that the expression of YAP and TEAD was increased in the Doxy group (**Figures 3A, B**). Then, MDSCs were divided into two main subsets of MDSCs, with immunosuppressive functions applied. It was discovered that the proportion of M-MDSCs increased more significantly compared to the M+T group, while the expression of G-MDSCs diminished in the DOXY treatment group (**Figure 3C**). Accordingly, the levels of iNOS and Arg-1 as expressed by M-MDSCs were also elevated (**Figures 3A, B**), as was the trend of NO expression (**Figure 3D**). By contrast, it was found out that the expression of ROS was reduced in the Doxy group, which is consistent with the decrease of G-MDSCs expression (**Figure 3E**). As confirmed by the detection of related cytokines in the cell supernatant, the levels of IL-6 and IFN- γ declined significantly in the Doxy treated group, indicating the effectiveness of YAP activation in reducing the level of pro-inflammatory cytokines, inducing the level of anti-inflammatory cytokines and confirming that Doxy intervention may suppress the inflammatory response (**Figure 3F**). In addition, it was further confirmed that the intervention of Doxycycline could inhibit the differentiation of Th17 cells to a significant extent, while increasing the proportion of Treg (**Figures 3G, H**). Accordingly, ELISA detection was also performed to confirm that Doxycycline treatment could contribute to increasing IL-10 and TGF- β, and suppressing the expression of IL-17 (**Figure 3F**). These results show that YAP activation can promote the proliferation of MDSCs, which is effective in inhibiting the production of pro-inflammatory cytokines, and in inducing the production of immunosuppressive cytokines.

### Glucocorticoids Relieves Liver Damage by Regulating Hippo Pathway to Promote MDSCs Proliferation and Suppress Immunity

**Figure 4:**
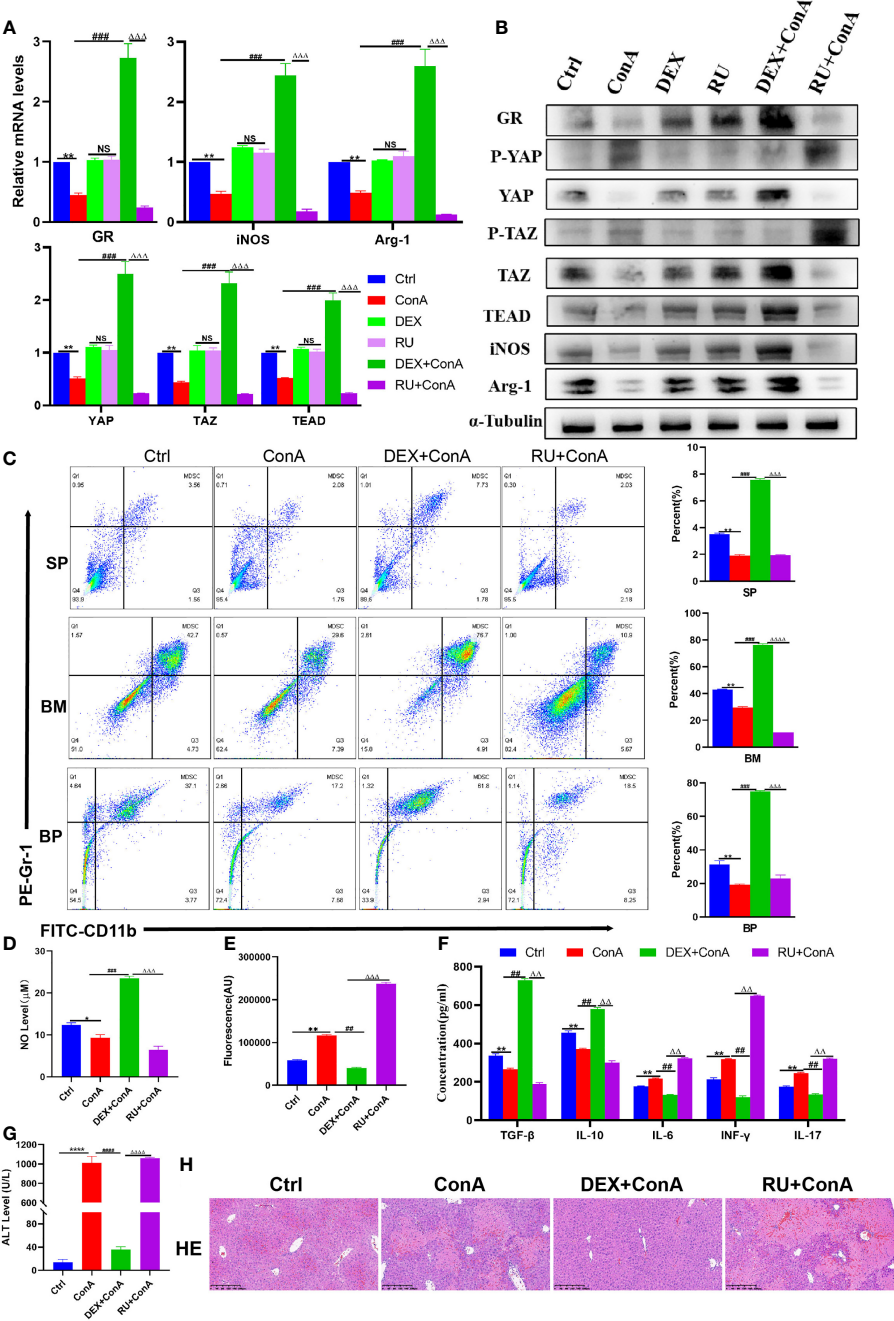
Glucocorticoids relieves liver damage by regulating Hippo pathway to promote MDSCs proliferation and suppress immunity. **(A)** The frequencies of MDSCs in spleen, bone marrow (BM) and peripheral blood (BP) (n=3). **(B)** Expression of related genes was evaluated by qRT-PCR in liver (n=3). **(C)** The relative expression levels of related protein in liver tissue were detected by Western blot (n=3). **(D)** No concentration in liver tissue (n=3). **(E)** Concentration of ROS in liver tissue (n=3). **(F)** The levels of related cytokines in liver tissue were detected by ELISA (n=3). **(G)** Serum ALT leve l(n=7). **(H)** Representative H&E histopathology staining of liver tissue (×100; scale bar: 200μm) (n=3). Data are expressed in mean ± SEM; *P < 0.05, **P < 0.01, ****P < 0.0001, control group versus Con A group; ##P < 0.01, ###P < 0.001, ####P < 0.0001, Con A group versus DEX +Con A group; △△P < 0.01, △△△P < 0.001, △△△△P < 0.0001, DEX +Con A group versus RU+ Con A group; NS, no significant difference.

At present, glucocorticoid is widely used in clinical practice as an effective drug for the treatment of AIH. Herein, the mice were pretreated with glucocorticoid and hormone inhibitor RU-486 to explore the potential mechanism of AIH treatment with glucocorticoid. Firstly, it was discovered that there was a decline in the expression of GR among Con A-treated mice. Then, it was further observed that there was not only an increase in the expression of YAP/TAZ-TEAD gene and protein in GR agonist group, but also a decrease in the expression of phosphorylated YAP and TAZ protein, indicating that the core effector protein of Hippo could activate the target genes after entry into the nucleus (**Figures 4A, B**). Given the previous experimental results, it can be confirmed that activating the coactivators downstream of the Hippo pathway is conducive to promoting the expression of MDSCs-related molecules. Furthermore, it was observed that GR activation increased the expression of Arg-1 and iNOS as well (**Figures 4A, B**). At the same time, it was discovered through flow cytometry that the percentage of MDSCs proliferation was higher in spleen, bone marrow and peripheral blood among the glucocorticoid intervention group than in the Con A group and inhibitor group (**Figure 4C**). In the GR intervention group, there was a significant increase in the level of MDSCs-mediated immunosuppression-related molecule NO, but a decline in the expression of oxidative stress-related factor ROS (**Figures 4D, E**). By further detecting and comparing the production of effector factors in the liver by ELISA, the results were obtained to suggest that the expression of IL-6, IL-17 and TGF- β was significantly inhibited, while that of IL-10 and INF-r related to Treg cell differentiation was promoted (**Figure 4F**). As confirmed by the liver function test of mice, DEX was effective in alleviating the liver impairment caused to AIH mice (**Figure 4G**). Morphological examination revealed that compared to the interface hepatitis in the AIH model group and RU group, there was a significant decline in the infiltration of inflammatory cells in the portal vein and around the portal vein and the necrotic area of hepatocytes among the DEX group than in the AIH mice and RU group (**Figure 4H**). In summary, it can be concluded that the DEX-activated GR is capable to regulate Hippo-YAP signal pathway, inhibit YAP phosphorylation, promote the expression of effector target genes, promote MDSCs proliferation, produce the effect of immunosuppression, and inhibit tissue oxidation and inflammatory damage, thus protecting the liver.

### Influence of GR on MDSCs and T Cells *In Vitro*

**Figure 5:**
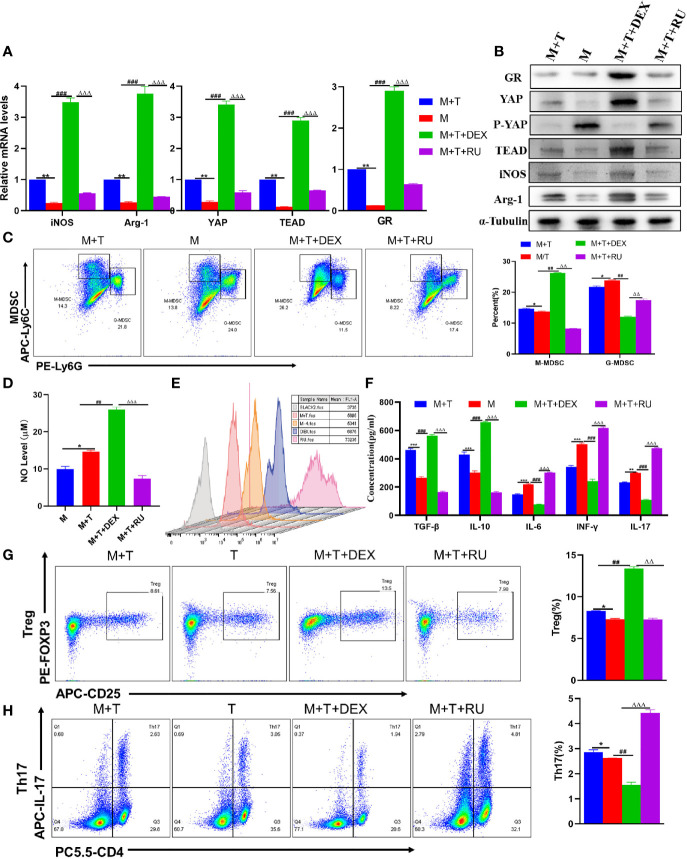
Influence of GR on MDSCs and T cells *in Vitro*. **(A)** Expression of related genes was evaluated using qRT-PCR; **(B)** Levels of related protein expression in MDSCs were analyzed by Western blotting; **(C)** Frequencies of M-MDSC (CD11b^+^Ly6C^high^ Ly6G^-^) and G-MDSC (CD11b^+^Ly6C^low^Ly6G^+^); **(D)** Detection of NO level in supernatant of cells; **(E)** ROS level measured in MDSCs using DCFH-DA staining; **(F)** The production of IFN-γ, TGF-β, IL-10, IL-6, IL-17 in the supernatants was measured by ELISA; **(G)** Frequencies of Tregs (CD4^+^CD25^+^Foxp3^+^); **(H)** Frequencies of Th17 (CD4^+^IL-17^+^); n=3. Data are expressed in mean ± SEM; *P < 0.05, **P < 0.01, ***P < 0.001, M/T group versus M+T group; ##P < 0.01, ###P < 0.001, M/T group versus M+T+DEX; △△P < 0.01, △△△P < 0.001, M+T+DEX versus M+T+RU group.

To verify the effect of GR activation on MDSCs and T cells, and whether GR activation is involved in the activation of YAP, DEX and RU were added to the MDSCs and T cell co-culture system, respectively. First of all, it was observed that with an increase in the expression of GR gene and protein, the expression of YAP and TEAD was also enhanced. In comparison, the expression of YAP and TEAD was reduced in the RU group, suggesting that the activation of GR is conducive to the transcription of YAP-TEAD. In the meantime, it was also found out that there was a rise in the expression of Arg-2 and iNOS (**Figures 5A, B**). According to flow cytometry analyses, glucocorticoid intervention played a role in promoting the differentiation of MDSCs into M-MDSCs rather than G-MDSCs in the co-culture system (**Figure 5C**). Meanwhile, DEX-amplified MDSCs showed a significant increase in NO production compared to baseline and RU-treated cells (**Figure 5D**). As revealed by ROS fluorescence quantitative detection, RU intervention enhanced ROS expression and exacerbated oxidative damage (**Figure 5E**). The trend of cytokine IL-6 expression was also demonstrated (**Figure 5F**). Furthermore, it was discovered that the differentiation of T cells into Treg showed an increasing trend while that of Th17 cells exhibited a decreasing trend in the glucocorticoid intervention group (**Figures 5G, H**). Accordingly, it was observed that the expression of IL-10 and TGF- β cytokines in DEX group was increased compared to the control group and RU-treated cell group, while that of IL-17 and INF-γ was reduced (**Figure 5F**). Therefore, it was speculated that glucocorticoid can activate GR, promote YAP-TEAD transcription, induce MDSCs proliferation and differentiation, enhance the differentiation of T cells to Treg, inhibit Th17 cell activation, and produce an immunosuppressive effect.

### GSS Can Play a Glucocorticoid-Like Effect and Protect AIH Mice

**Figure 6:**
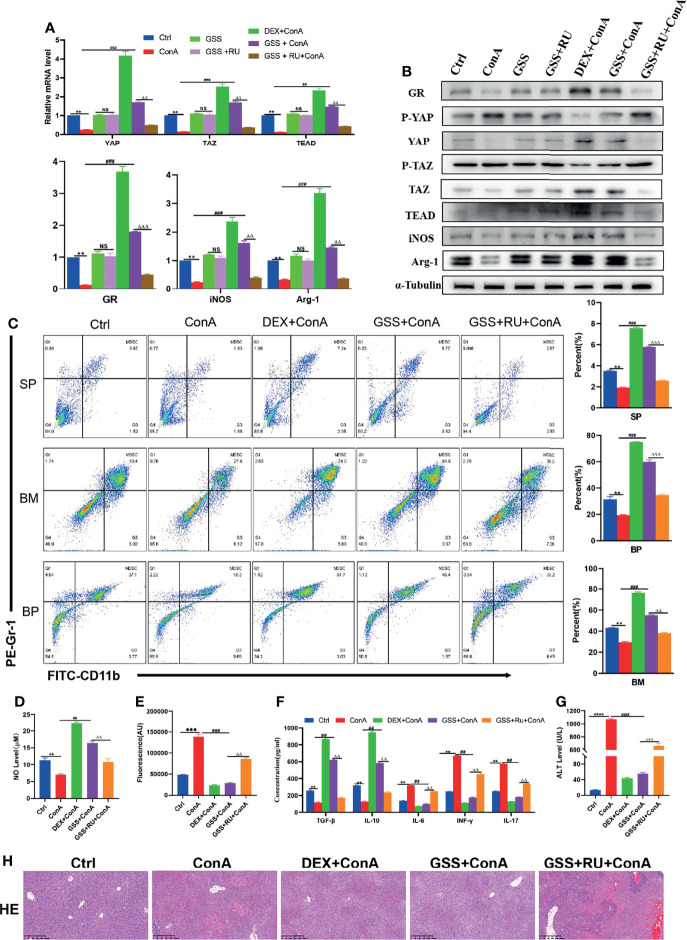
GSS can play a glucocorticoid-like effect and protect AIH mice. **(A)** The frequencies of MDSCs in spleen, bone marrow(BM) and peripheral blood (BP) (n=3). **(B)** Expression of related genes was evaluated by RT-PCR in liver (n=3). **(C)** The relative expression levels of related protein in liver tissue were detected by Western blot (n=3). **(D)** No concentration in liver tissue (n=3). **(E)** Concentration of ROS in liver tissue (n=3). **(F)** The levels of related cytokines in liver tissue were detected by ELISA (n=3). **(G)** Serum ALT level (n=7). **(H)** Representative H&E histopathology staining of liver tissue (×100; scale bar: 200μm) (n=3). Data are expressed in mean ± SEM; **P < 0.01, ***P < 0.001, control group versus Con A group; ##P < 0.01, ###P < 0.001, ####P < 0.0001, Con A group versus DEX +Con A group& GSS +Con A group; △△P < 0.01, △△△P < 0.001, GSS +Con A group versus GSS + RU+ Con A group.

Previously, it was confirmed that GSS is a GR agonist (26). Therefore, it was speculated that GSS can play a glucocorticoid-like effect on AIH by activating GR. To prove the GR-dependent therapeutic effect of GSS, the mice were pretreated with DEX, GSS and GSS+ RU, respectively. First of all, it was found out that the expression of GR gene and protein was improved in GSS-treated mice, which could be suppressed by the combination of RU, indicating that GSS is a glucocorticoid receptor agonist (**Figures 6A, B**). Then, it was discovered that GSS intervention could play a similar role to DEX in promoting the expression of YAP/TAZ-TEAD and inhibiting protein phosphorylation (**Figures 6A, B**). Furthermore, it was observed that GSS treatment increased the expression of Arg-1 and iNOS as well, suggesting that GSS had an effect on the expression of MDSCs (**Figures 6A, B**). These can be verified by flow cytometry to analyze the changes in MDSCs proliferation ratio of MDSCs in spleen, bone marrow and peripheral blood. According to the results, the proportion was higher in DEX group and GSS group (**Figure 6C**). As suggested by the detection of NO level in liver tissue, GSS also produced a glucocorticoid-like effect and increased the level of NO (**Figure 6D**). In comparison, GSS produced an anti-inflammatory effect, as confirmed by the expression level of ROS detected in liver tissue and that of IL-6 detected in peripheral serum (**Figure 6E**). In addition, there were other immune-related cytokines detected in peripheral serum. According to the results, the expression of IL-17 and TGF- β was significantly inhibited, while that of IL-10 and INF-γ in GSS group was increased (**Figure 6F**). The liver enzyme level and liver histopathology were detected to verify the hepatoprotective effect of GSS. It can be seen clearly that GSS reduced the level of ALT in mice to a significant extent (**Figure 6G**). In addition, the pathological examination of liver tissue revealed that the necrosis of hepatocytes decreased, the infiltration of inflammatory cells was insignificant, and the structure of liver tissue in GSS pretreated mice was undamaged (**Figure 6H**). After the intervention of RU, however, it was observed that the immunosuppressive, anti-inflammatory and antioxidant effects of GSS were reversed (**Figures 6G, H**). Therefore, it can be concluded that GSS is a GR activator that can produce a glucocorticoid-like effect on AIH mice.

### GSS Regulates the Expression of YAP-TEAD by Activating Glucocorticoid Receptors, and Has an Impact on the MDSCs and T Cell Co-Cultivation System

**Figure 7:**
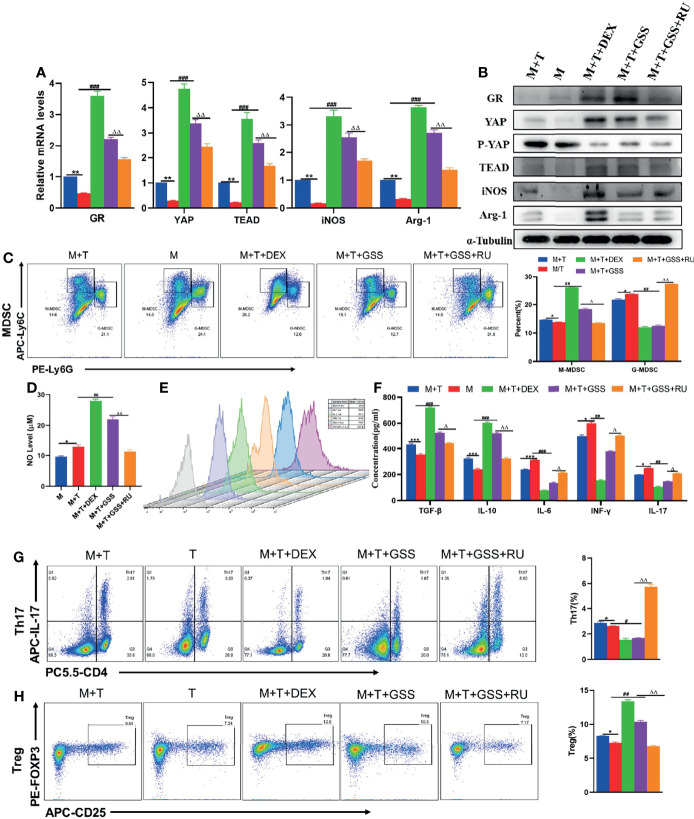
GSS regulates the expression of YAP-TEAD by activating glucocorticoid receptors, and has an impact on the MDSCs and T cell co-cultivation system. **(A)** Expression of related genes was evaluated using qRT-PCR; **(B)** Levels of related protein expression in MDSCs were analyzed by Western blotting; **(C)** Frequencies of M-MDSC (CD11b^+^Ly6C^high^ Ly6G^-^) and G-MDSC (CD11b^+^Ly6C^low^Ly6G^+^); **(D)** Detection of NO level in supernatant of cells; **(E)** ROS level measured in MDSCs using DCFH-DA staining; **(F)** The production of IFN-γ, TGF-β, IL-10, IL-6, IL-17 in the supernatants was measured by ELISA; **(G)** Frequencies of Tregs (CD4^+^CD25^+^Foxp3^+^); **(H)** Frequencies of Th17 (CD4^+^IL-17^+^); n=3. *P < 0.05, **P < 0.01, ***P < 0.001, M/T group versus M+T group; #P < 0.05, ##P < 0.01, ###P <0.001, M/T group versus M+T+DEX group & M+T+GSS group; △P < 0.05, △△P < 0.01, M+T+GSS group versus M+T+GSS+RU group.

In order to further verify that GSS can also produce a glucocorticoid-like effect *in vitro* and a regulatory effect on MDSCs and T cells, DEX, GSS and GSS+RU were added to MDSCs and T cell co-culture system, respectively. Through the detection of qRT-PCR and WB, it can be found out that the expression of GR gene and protein was enhanced after the intervention of GSS and DEX, thus confirming that GSS could also activate GR *in vitro*. Differently, the expression of GR was reduced in the combined RU intervention group, which indicates that the activation of GR by GSS could be suppressed by GR inhibitors (**Figures 7A, B**). In the meantime, it was also found out that the expression of YAP and TEAD was also promoted in DEX and GSS intervention groups, suggesting that the activation of GR by GSS can be effective in promoting the transcription of YAP-TEAD (**Figures 7A, B**). Likewise, there was an increase in the expression of Arg-1 and iNOS genes and proteins as observed in GSS group (**Figures 7A, B**). According to the results of flow cytometry, in comparison with the control group and RU-treated group, the intervention of GSS could promote the differentiation of MDSCs into M-MDSCs, rather than G-MDSCs, which is similar to DEX (**Figure 7C**). In parallel, GSS intervention can also give rise to NO (**Figure 7D**). As revealed by ROS fluorescence quantitative analysis, GSS suppressed the fluorescence expression of ROS (**Figure 7E**). The trend of IL-6 expression in the supernatant was found identical to that of ROS (**Figure 7F**). Then, it was found out that GSS promoted the differentiation of T cells into Treg, while inhibiting the differentiation of Th17 cells (**Figures 7G, H**). Accordingly, the detection of related cytokines suggested that the expression of IL-10 and TGF- β cytokines was increased, while the expression of IL-17 and INF-γ was reduced in the GSS intervention group (**Figure 7F**). Therefore, it was speculated that GSS could also activate glucocorticoid receptors *in vitro* and further regulate MDSCs and T cells by regulating YAP-TEAD.

## Discussion

As the pathogenesis of autoimmune hepatitis remains unclear, it is unlikely for the existing treatment strategies to meet the expectation of all patients. Extracted from Chinese herbal medicine, several compounds have been shown to have therapeutic effects on AIH, such as resveratrol, paeony polysaccharide (PRAP) and pomegranate peel extract (PoPx), all of which can mitigate the immune impairment caused to experimental autoimmune hepatitis mice by suppressing liver immune response (35–37). In recent years, there have been widespread attention paid to the effects of ginsenosides on anti-inflammation, regulating immunity, anti-tumor and protecting liver *in vivo* and *in vitro*. However, there is still limited literature on ginsenosides for the treatment of AIH and its mechanism.

Con A-induced liver impairment belongs to a cytokine-dependent model of acute liver injury. As demonstrated by Ye et al., the levels of ALT, AST, LDH and various inflammatory cytokines, including TNF- α, IFN-γ and IL-6, showed an increasing trend in the Con A-treated mice with 20mg/kg body weight for 12 hours, which is similar to that of clinical AIH patients (38). By evaluating the mice in the AIH model group, it was confirmed that the level of serum ALT increased significantly after Con A treatment. Besides, liver tissue H&E staining indicated focal hepatocyte necrosis, while extensive monocyte infiltration was observed in the portal vein area and around the central vein. At the same time, the levels of IL-6 and INF-γ in peripheral serum of Con A-treated mice were higher compared with the normal group. After the pretreatment with GSS, the level of liver enzymes and ROS in mice diminished, the pathology of liver tissue returned to normal, and the level of pro-inflammatory cytokines declined as well, thus confirming that GSS pretreatment can alleviate the liver impairment induced by Con A to a significant degree.

In recent years, it has been demonstrated that innate immunity not only drives the acquired immune response but also facilitates the development of autoimmune diseases, which may be essential for the treatment of diseases (39, 40). MDSCs are known as an important part of the innate immune system. In recent studies, it has been suggested that, MDSC is highly effective in inhibiting various T cell functions (41). Con A-induced hepatitis is a highly specific T cell activated liver injury model. Similar to other autoimmune liver diseases, AIH is also associated with the abnormalities in the Th17 pathway, while the high frequencies of Th17, IL-17 and IL-23 are observable in the liver and serum (15, 42). According to Liberal et al., the hypothesis has been evaluated that regulatory T cells are deficient in the patients with AIH, which may be related to the low expression of FOXP3. Besides, there are also some differences in regulatory T cells among AIH patients, which is related to the inability to inhibit the production of IFN- γ (42). Another mechanism is the response of effector T cells to Treg that may be damaged in AIH (43). Currently, it has been confirmed that functional Treg cells play a vital role in inhibiting the production of IL-10 and TGF-β during the development of immune tolerance (44, 45). By detecting the peripheral blood of mice, it was confirmed that there was not only a sharp rise in the levels of serum INF- γ and IL-17 in AIH model mice, but also a decline in the levels of IL-10 and TGF- β, which could mediate immunosuppression. In fact, the accumulation of MDSCs involves two processes, expansion and activation, both of which are controlled by different cytokines and signal transduction pathways (41). Produced in acute or chronic infection, trauma or tumor microenvironment, a variety of cytokines can promote the accumulation of immature myeloid cells (IMCs) in the lesion site, thus forming cells with immunosuppressive function, known as MDSCs. It involves GM-CSF, M-CSF, G-CSF, IL-6, VEGF and other factors, with the signal mainly transmitted through STAT3 and STAT5. By stimulating myelopoiesis and inhibiting myeloid cell differentiation, STAT3 regulates the expansion of MDSCs, which increases the production of ROS (46). However, this signal alone is still insufficient to cause the accumulation of MDSCs. Requiring a second activation signal, MDSCs are characterized by the up-regulation of arginase, NO, production of immunosuppressive cytokines and so on. This signal can be released by such pro-inflammatory molecules as INF-γ, IL-1β, IL-13, TLR ligands and so on. These factors activate not only STAT6 and STAT1, but also TLR-mediated nuclear factor kappa B (NF-κB) activation, thus leading to the activation of MDSCs, and then the up-regulation of inducible nitric oxide synthase and arginase. In the meantime, this result in the production of inhibitory cytokines such as TGF- β (47). MDSCs suppress T cell function through a variety of mechanisms, including the high levels of arginase activity and the production of NO and ROS. These main pathways are linked to different subsets of MDSCs. More specifically, ROS is connected to G-MDSCs, while arginase and iNOS are connected to M-MDSCs (31). Also, there are some studies confirming that to inhibit NLRP3 by preventing the production of ROS can help alleviate the damage of AIH (48). In addition, iNOS can promote both inflammation and immunosuppression. Some studies have suggested that iNOS is an acute and chronic inflammatory mediator that can further promote the production of IL-1 β by enhancing the production of inflammatory bodies such as NLRP3 (49). At the same time, however, it has been confirmed in iNOS studies that iNOS can also produce an immunosuppressive effect, which is closely related to MDSCs (50). The inhibitory effect of MDSCs is related to the metabolism of L-arginine (51), which refers to the substrate of two enzymes. One is the iNOS that produces NO and the other is the arginase that converts L-arginine into urea and L-ornithine. MDSCs expresses high levels of arginase and inducible nitric oxide synthase simultaneously. Besides, the immediate effect of these two enzymes on inhibiting T cell functions has already been confirmed (52). In our study, it was confirmed that the differentiation of MDSCs was inhibited in AIH mice. After MDSCs infusion, there were signs observed that the immune injury and oxidative inflammatory injury suffered by mice were alleviated. *In vitro* experiments, it was also confirmed that MDSCs acted as an immunosuppressant after T cells were co-cultured with MDSCs. Furthermore, it was confirmed through the intervention of GSS in mice that the proportion of MDSCs increased, as did the levels of Arg-1, iNOS and NO, despite a reduction in the expression of ROS. *In vitro*, GSS intervention promoted the proliferation of M-MDSCs, instead of G-MDSCs. In addition, the expression of Treg and related cytokines IL-10 and TGF- β exhibited an increasing trend after GSS treatment, while that of Th17, INF- γ and IL-17 was reduced. In conclusion, it was confirmed that GSS triggered T immunosuppression by promoting MDSCs, especially M-MDSCs.

It has been shown in recent studies that the activation of Hippo-YAP signal pathway can promote the differentiation and accumulation of MDSCs (22). In Kras:p53 mutated pancreatic ductal cells, YAP initiates the expression and secretion of various cytokines/chemokines, thus enhancing the differentiation and accumulation of MDSCs both *in vivo* and *in vitro (* 33). In addition, the proportion of MDSCs declined after the pharmacological inhibition of YAP1 *in vivo* in the mouse model infected with Chlamydia trachomatis (53). However, it remains debatable whether Hippo-YAP is involved in the pathogenesis of AIH. In our experiment, it was confirmed that the expression of YAP/TAZ-TEAD, as the core effector of Hippo-YAP, diminished in Con A-induced AIH mice. Through the intervention of YAP activator in mice and MDSC and T cell co-culture system, it was discovered that there was not only an increase in the proportion of MDSCs and the expression of related downstream molecules increased, but also a decline in the differentiation of immunosuppressive T cells and inflammatory cytokines, implying the involvement of Hippo-YAP signal pathway in the progression of AIH disease. Furthermore, it was also observed that the intervention of GSS could stimulate the up-regulation of transcriptional coactivator YAP/TAZ-TEAD, thus producing an immunosuppressive effect.

The current practice of AIH treatment is prednisolone alone or in combination with azathioprine, both of which are immunosuppressants (54). It is widely known that GC can have such biological effects as immunosuppression and anti-inflammation by regulating GR. However, there is still no study on how to regulate the immune microenvironment effect of GR by regulating downstream signal pathways in the treatment of AIH. In some studies, it has been shown that GC can activate the proliferation of YAP and drug-resistant tumor cells in breast cancer cells (55). YAP and TAZ can be activated by means of dexamethasone intervention, so as to produce a neuroprotective effect (56). In our experiment, it was also confirmed that the intervention of GC can not only regulate the activity of Hippo-YAP signal pathway but also exert an immunosuppressive effect on the immune microenvironment constructed using MDSCs and T cells, such as promoting the differentiation of MDSCs into M-MDSCs and T cells into Treg rather than Th17. In addition, our previous studies have confirmed that GSS can produce glucocorticoid-like effects (26). By further comparing the expression levels of downstream pathways and related molecules between GSS group and GC group, it can be confirmed that GSS could activate GR and further up-regulate the expression of YAP/TAZ-TEAD, so as to regulate the proliferation and differentiation of MDSCs and T cells, such as expanding functional MDSCs, increasing the expression of IL-10, TGF- β and Tregs proportion, reducing the expression of INF- γ and IL-17, as well as inhibiting the production of ROS and IL-6.

To sum up, this study explored the therapeutic effect of GSS on experimental autoimmune hepatitis and its mechanism, which provides a theoretical basis for GSS as a solution to the treatment of AIH. According to the results of this study, GSS could activate GR, regulate Hippo-YAP signal pathway, and activate innate immunity, thus improving the proliferation of MDSCs, enhancing its differentiation into M-MDSCs and further promoting the secretion of Arg-1, iNOS and NO. This has effect on adaptive T cell immunity, which increases the percentage of Treg, reduces the percentage of Th17, up-regulates the production of immunosuppressive cells, and suppresses the production of pro-inflammatory cytokines. Ultimately, the activity of the disease is reduced. In conclusion, these findings demonstrate that the Hippo-YAP signaling pathway, the immune microenvironment balance of MDSCs and T cells can serve as a possible key target for AIH therapy, which provides new insights into the therapeutic potential of GSS in the treatment of AIH.

## Data Availability Statement

The original contributions presented in the study are included in the article/supplementary material. Further inquiries can be directed to the corresponding author.

## Ethics Statement

The animal study was reviewed and approved by Shanghai Municipal Hospital of Traditional Chinese Medicine.

## Author Contributions

KZ performed data acquisition and analysis, and wrote the manuscript. JL, SZ, YZ, and others conducted experimental operation and data collection and analysis. YL conceived the study design and is the guarantor of the article. All authors contributed to the article and approved the submitted version.

## Funding

This work was supported by the National Natural Science Foundation of China (NO. 81573775; NO. 81873157) and the Postgraduate Innovation Training Special Project (Y2020020).

## Conflict of Interest

The authors declare that the research was conducted in the absence of any commercial or financial relationships that could be construed as a potential conflict of interest.

## Publisher’s Note

All claims expressed in this article are solely those of the authors and do not necessarily represent those of their affiliated organizations, or those of the publisher, the editors and the reviewers. Any product that may be evaluated in this article, or claim that may be made by its manufacturer, is not guaranteed or endorsed by the publisher.
